# Female condom acceptability and use amongst young women in Botswana

**DOI:** 10.4102/curationis.v41i1.1887

**Published:** 2018-09-20

**Authors:** Moitlamo Mokgetse, Mokholelana M. Ramukumba

**Affiliations:** 1 Ministry of Health, Gaborone, Botswana; 2 Department of Health Studies, University of South Africa, South Africa

## Abstract

**Background:**

The female condoms are a barrier method of contraception. The FC1 female condom is made from soft thin plastic called polyurethane and has been replaced by FC2 female condom, which is made of synthetic latex. The female condom is worn inside the vagina and acts as a barrier to prevent semen getting to the womb. It is about 75% – 82% effective with normal use. When used correctly all of the time, female condoms are 95% effective. Despite evidence of its effectiveness, the use of the female condom has continued to face resistance from both women and men.

**Objectives:**

The objectives of this study were to determine clients’ level of knowledge of female condom, identify predominant methods of contraception, examine young women’s views regarding the female condom and identify barriers to the use of female condoms by young women.

**Method:**

A simple random sampling approach was used to recruit young women between 15 and 34 years in Jwaneng, Botswana. Data were collected using self-administered structured questionnaire from women accessing healthcare services in the three health facilities in 2015. Descriptive statistics, exploratory factor analysis and the chi-square test techniques were applied using the Statistical Package for the Social Sciences (SPSS) statistical programme version 23 for Windows to analyse data.

**Results:**

The findings based on factor loadings show low utilisation of the female condom and highlights the significant challenges about the material, size, shape and timing of insertion. Respondents had stronger views on the structure of the condom. There was no significant relationship between attitude and method of contraception.

**Conclusion:**

Acceptability of the female condom involves complex factors such as women position and decision-making power in a relationship, attitude and knowledge. Attitude, knowledge and power relations play a role in the extent to which women would want to try or use it. Various strategies need to be developed to effectively promote the female condom amongst young women.

## Introduction

Human immunodeficiency virus (HIV) and/or acquired immune deficiency syndrome (AIDS) is a global public health problem that continues to affect millions of people around the world, especially in developing countries. The joint United Nations Programme on HIV/AIDS (UNAIDS) Global Report (UNAIDS 2015) shows that sub-Saharan Africa accounts for 66% of the global total of new HIV infections. Women account for more than half the total number of people with HIV. Adolescent girls and young women continue to experience elevated HIV risk and vulnerability. Women are disproportionately affected by the epidemic and account for 31% of new infections and such rates are three times higher than in men. Tobin-West et al. (2014) revealed that at the end of 2014, an estimated 36.9 million people were living with HIV globally, of which 34.3 million were adults and women accounted for 17.4 million. Botswana continues to have one of the highest rates of HIV prevalence in the world. Although the rate of new HIV infections has dropped, the prevalence rate remains high amongst certain populations, such as young persons with an estimated 23% of 15- to 49-year olds being HIV infected (Sarumi & Strode 2015). In Jwaneng Township, women continue to record high HIV prevalence rate of 16.1% as compared to 8.3% amongst men (Government of Botswana 2013).

Literature indicates various factors related to women and HIV and/or AIDS infection, and poverty tends to increase their vulnerability. Korndoerfer (2014) cited lack of knowledge of how to prevent infection as well as financial constraints. Power relations between men and women in a relationship were also an issue. Young women often have no choice regarding unprotected sex, thus increasing the risk of infection (Korndoerfer 2014). Since the beginning of the AIDS epidemic, condoms have been promoted as most efficient. In 1993, the United States Food and Drug Administration approved the female condom as a safe and efficient contraceptive and protective device (Peters, Van Driel & Jansen 2010; Tobin-West et al. 2014). In response to women’s disproportionate disease burden, much effort in HIV prevention research has been focused on the identification of female-initiated barrier methods (Montgomery et al. 2012). Female condom is one of the family planning methods that is being promoted as the only female-initiated prevention device that offers dual protection against pregnancy and sexually transmitted infections (STIs) including HIV and/or AIDS. The female condom can be inserted up to 8 hours before sexual intercourse. Moreover, because it is worn by women, its use may increase women’s control over their reproductive health (Koster et al. 2015). A number of female condom products are currently available to consumers whilst others are still in development. These products vary in appearance, design, material and regulatory status (Peters et al. 2010).

Despite evidence of its effectiveness, the use of female condom has continued to face resistance from both women and men. Kaelo and Malema (2014) posit that despite extensive condom promotion efforts, condom use in sub-Saharan Africa remains very limited. Ahmed et al. (2012) argue that the female condom must be acceptable to both men and women in order to prevent STIs and unwanted pregnancies. Furthermore, they explained that women may be able to negotiate use of the female condom more easily than the male condom, giving them potentially more power to protect themselves in a sexual relationship. About 79% of university students in Rwanda were aware of the female condom and the fact that it can prevent unwanted pregnancies and STIs though its use was very low as only 24% of the students knew how to use it (Valens & Joseph 2013; Wang et al. 2014). Bogale, Boer and Seydel (2010) found that women who use condoms have a significantly higher attitude, perceived a significantly higher level of self-efficacy, felt more vulnerable to HIV infection and perceived condoms to be more effective in preventing STIs. Mantell et al. (2011) documented challenges with female condom availability, affordability and ease of insertion. Tobin-West et al. (2014) cited discordance between awareness and the preventive practice of female condom use.

The Government of Botswana has developed health promotion initiatives to encourage the use of the female condom and to educate the public about female condom use. Health promotion campaigns, which include the use of multimedia (newspapers, radio spots and television commercials and use of billboards) and art or drama, have been introduced throughout the country. Education for service providers and sensitisation of community members is carried out during workplace wellness campaigns as well as health expositions (own experience). Public health preventive measures taken by the government ensure that condoms are accessible and available to consumers.

## Problem statement

It is almost a decade since the female condom was introduced in Botswana as the only female-initiated method that offers dual protection for STIs, HIV and/or AIDS and pregnancy. In 2010, the United Nation Population Fund (UNPFA) provided 840 million male condoms and 9.8 million female condoms, mostly to sub-Saharan Africa (United Nations Population Fun [UNFPA] 2011). In Jwaneng, HIV prevalence amongst women is 16.1% as compared to 8.3% amongst men (Government of Botswana 2013). Data from health facilities in Jwaneng showed that from January to December 2013, a total of 86 890 male condoms were distributed as compared to 811 female condoms (Government of Botswana 2013). The factors related to this low distribution are unknown. Therefore, it was important to study the acceptability of the female condom as measured by the extent of use amongst young women in Jwaneng. The study intended to find answers to the following questions:

- What is the level of knowledge amongst young women regarding female condoms?
- What are the predominant methods of contraception?
- How do young women view female condoms?
- What are the barriers to the use of female condoms?

## Definition of concepts

### Knowledge

Knowledge in this study refers to awareness of female condoms measured by exposure to health promotion.

### Acceptability

This study defines acceptability as the potential users’ level of female condom use as a method to prevent unintended pregnancies, STIs and HIV and/or AIDS.

### Views

Refers to attitude, opinions and beliefs of young women regarding the female condom.

### Barriers

Situations or problems that prevent or hinder young women from using female condoms.

### Young women

Women between the ages of 15 and 34 years old, residing in Jwaneng, accessing family planning services in the clinics and exposed to female condom health promotion.

## Methods

A quantitative descriptive study was conducted using 95 young women aged 15–34 years residing in Jwaneng. A sample framework was created from the family planning register and a random selection of sample with replacement was utilised as they attended the sexual reproductive health clinic service (Brink, Van der Walt & Van Rensburg 2014). The inclusion criteria were age and number of times women used the family planning service. A frequency of six was deemed acceptable, as it implied the women were exposed to female condom health education six times. The total population for the study was 271. The sample size was calculated using Raosoft online sample size calculation, using the margin error of ± 5%, 95% confidence level, with 50% distribution yielded a sample of 136. The data cleaning and validation process eliminated all cases (participants) that had not answered at least one question and retained only cases that answered all questions. The final sample size after data cleaning was 95.

A self-administered questionnaire was used to collect data. The questionnaire was adapted from Chirwa (2011) to determine the extent of female condom acceptability. Scale items in the questionnaire consisted of statements that needed to be rated on various Likert scales. The tool was distributed to the women and was completed during six sessions in the three health facilities. The information leaflet stated that participation was voluntary and participants could withdraw at any stage. Measures were the demographic data, knowledge and use of the female condom, general views about the female condom, and possible recommendations.

Cronbach’s alpha coefficient was statistically measured to assess the extent to which identical responses could be obtained if the same questions were posed to the same respondents several times (Brink et al. 2014). To improve scale reliability, some items were eliminated from the instrument. The Cronbach’s alpha coefficient value (α = 0.741) for the finally selected items exceeded the minimum acceptable (α = 0.7) condition of total scale reliability. The statistical validity of the items of the research instrument was undertaken using exploratory factor analysis data reduction technique. Factor analysis was undertaken to focus on a set of factors that accounted for most of the observed variance in the data set with regard to acceptability of the female condom amongst the target respondents. In undertaking the analysis, the sampling adequacy of survey items of the research instrument was measured based on the Keiser–Meyer–Olkin Measure of Sampling Adequacy (MSA) criterion.

The demographic information was used as a control variable. Descriptive and inferential statistical analysis using Statistical Package for the Social Sciences (SPSS) software version 23 for Windows was used to calculate, summarise and describe the acceptability of female condom use. The chi-square non-parametric test of statistical significance for bivariate tabular analysis was applied to examine whether there is an existing association between the contraception method and general views about the female condom extracted through factor analysis.

### Ethical considerations

The Health Research and Development Division of Ministry of Health approved the study. The Department of Health Studies Ethics Committee, University of South Africa, issued the ethical clearance certificate (HSHDC/355/2014). Consent was obtained from the selected participants after the Head of Jwaneng District Health Management Team granted permission.

## Results

An examination of the profile of the respondents showed that the majority at 41.05% were between 25 and 29 years, which was also the median age group. All respondents were above 18 years. The highest percentage of 83.16% were single women, followed by 14.74% being married women. The largest proportion of about 43.16% respondents indicated that they were not employed, whilst approximately 31.58% of the respondents were permanently employed during the time the survey was conducted. Though the majority of these women were unemployed, they had attained a higher level of education (secondary and tertiary level). Of the 95 respondents, 48.42% reported that they possessed tertiary educational qualifications whilst approximately 46.32% of the respondents reported that they had secondary educational qualifications up to the time the survey was conducted. The majority were Christians at 96%. The most common method of protection used was the male condom at 65.26%, followed by oral contraceptives and injectable at 10.53% each (see Table 1).

**TABLE 1:** Socio-demographic characteristics of respondents.

Characteristics	Frequency (*n*)	Percentage (%)
**Age (*n* = 95) years**		
15–19	7	7.37
20–24	20	21.05
25–29	39	41.05
30–34	29	30.53
**Marital status (*n* = 95)**		
Single	79	83.16
Married	14	14.74
Separated	1	1.05
Cohabitating	1	1.05
**Employment status (*n* = 95)**		
Not employed	41	43.16
Self-employed	13	13.68
Temporarily employed	11	11.58
Permanently employed	30	31.58
**Level of education (*n* = 95)**		
Never studied	2	2.11
Non-formal education	1	1.05
Primary	2	2.11
Secondary	44	46.32
Tertiary	46	48.42
**Religion (*n* = 95)**		
Christianity	91	96
Muslim	2	2.11
Hindu	1	1.05
Non-affiliation	1	1.05
**Method of contraception (*n* = 95)**		
Natural method	6	6.32
Male condom	62	65.26
Female condom	4	4.21
Oral contraceptives	10	10.53
Injectable	10	10.53
Norplant	3	3.16

Knowledge in this study refers to awareness of female condoms measured by exposure to health promotion. All of the respondents were exposed to female condom health promotion and had seen it. Approximately 25.26% indicated that their main source of information was a pamphlet or banner, whilst about 13.68% reported that their main source of information was television. The majority, 44.21%, reported that their main source of information was the health facility. Although all had heard of a female condom before, almost 90.53% reported that they had seen it (see Table 2).

**TABLE 2:** Knowledge and source of information about female condom.

Variable	Frequency (*n*)	Percentage (%)
**Ever heard of the female condom (*n* = 95)**		
Yes	95	100.00
No	0	0.00
**Main source of information (*n* = 95)**		
Conference or workshop	5	5.26
Facebook or Twitter	1	1.05
Pamphlets or banners	24	25.26
Health facility	42	44.21
Friends	3	3.16
Radio	4	4.21
Television	13	13.68
Others	3	3.16
**Ever seen female condom (*n* = 95)**		
Yes	86	90.53
No	9	9.47

### Views regarding the female condom

This section used the scale of 1–5. Based on the approximate mean statistics, the respondents 100% (*n* = 95) surveyed on average agreed (mean = 4) that the use of female condoms requires cooperation of both partners, that a condom gives more options for protection and that the female condom is made of stronger material. Furthermore, respondents strongly agreed (mean = 5) that insertion of a female condom seems to be a hassle and it looks odd. The computed standard deviation statistics show that responses from participants demonstrate low variation from the mean; hence, the responses of the participants were less spread out (see Table 3).

**TABLE 3:** General views about female condom.

Variable	Statistic (*N*)	Mean	Standard error	Standard deviation
Use of a female condom needs the cooperation of both partners	95	4.17	0.113	1.098
The female condom gives more options for protection	95	4.14	0.105	1.027
A female condom is made of stronger material	95	4.11	0.088	0.856
Insertion seems to be a hassle	95	4.63	0.064	0.620
Female condom looks odd	95	4.63	0.065	0.637
Valid *N* (list wise)	95	-	-	-

### Barriers to the use of female condom

Approximately 38.94% of the respondents agreed that the material of female condoms could make them difficult to use, whilst nearly 17.89% strongly agreed with the same perception. About 96.84 indicated that the device was too big and too long, whilst 20% felt that information on the female condom was not readily available. A small number 4.21% believed that the device can reduce enjoyment, as it needs to be inserted correctly and way before the onset of sexual activity. Nearly 56.84% were of the perception that the female condom was not promoted enough.

### Relationship between method of contraception and general views regarding female condoms

There were no statistically significant associations between contraception method and general views about the female condom as shown in Table 4. The null of association between differences in each of the items specified and contraception method is rejected for all constructs.

**TABLE 4:** Relationship between method of contraception and general views regarding female condom.

Item	Pearson’s χ^2^ value	df	Asymptotic significance (two-sided)	Decision
Use of a female condom needs the cooperation of both partners.	23.280	20	0.275	Reject H_0_
The female condom gives more options for protection.	22.330	20	0.323	Reject H_0_
A female condom is made of stronger material.	14.509	20	0.804	Reject H_0_
Insertion seems to be a hassle.	6.670	15	0.966	Reject H_0_
Female condom looks odd.	7.213	15	0.951	Reject H_0_

df, degrees of freedom.

Based on results in Table 4, the null of the association between differences in each of the items specified and contraception method is rejected for all constructs. The results are as follows: χ^2^ (0.05; 20) = 23.28, *p* = 0.275 for cooperation in condom use; χ^2^ (0.05; 20) = 22.33, *p* = 0.323 for condom giving more options; χ^2^ (0.05; 20) = 14.509, *p* = 0.804 for female condom made of stronger material; χ^2^ (0.05; 15) = 6.670, *p* = 0.966 for insertion seems to be a hassle; and χ^2^ (15) = 7.213, *p* = 0.951 for the look of the female condom. All suggest that there were no statistically significant associations between contraception method and the distinct specified items.

## Discussion

The study results showed that the use of the female condom was low across all age groups, educational level and marital status. Kaelo and Malema (2014) support these findings and explain that despite extensive condom promotion efforts, condom use in sub-Saharan Africa remains very limited. An overwhelming 83.16% of the women were single, which may indicate some level of autonomy in reproductive decision-making. The rate of unemployment was 43.16%; this might mean that the women were dependent on others for a living, thus reducing the chance of using the female condom as it is assumed that they may have low bargaining powers. Maticka-Tyndale (2012) reports that across multiple diverse cultural groups, men in sub-Saharan Africa control sex and condom use. Gender inequality and the issue of power relations in which men play dominant roles in decision-making in the family exert too much influence on women‘s decisions (Vouking, Evina & Tadenfok 2014).

The majority, 94.74%, of the respondents had secondary and tertiary education. The Botswana AIDS Impact Survey IV (BAIS IV) study also found that 85.9% of the adult population aged 15 years and above in Botswana were estimated to be literate (Government of Botswana 2013). Given the high number of women with secondary and tertiary education, one would have expected the female condom uptake to be higher because they would have higher access to any form of information about the female condoms. The use of male condoms by majority, 65.26%, might be indicative of awareness and knowledge of preventive benefits. The fact that the women partners used condoms, it could be assumed that men may be amenable to a discourse on female condom. In support, Mantell et al. (2011) found that men were open to the idea of a female condom but they are an untapped audience.

All the respondents reported that they had heard of a female condom; the main source of the health promotion was the clinic and health promotion material. The study assumed women had good access to information. Therefore, they had knowledge. Yet, only 4.21% used it. Similarly, Wang et al. (2014) found no correlation between knowledge and use of the female condom in China. Their study revealed that 26.9% of women had previously heard of the female condom, 10.3% understood its function and method of use but only 0.1% used it. This shows that in general, knowing about the female condom does not necessarily translate to its use. Tobin-West et al. (2014) also found discordance between awareness and preventive practice of female condom use.

Women presented various views regarding the female condom. Need for cooperation and more options open for women to use preventive measures, the strength of the condom material had average scores (mean = 4). Meanwhile, respondents strongly agreed (mean = 5) that insertion could be tedious and they were not impressed with the odd look of the condom. The respondents indicated that use the of the female condom necessitated cooperation of both partners, which may be indicative of some power dynamics between men and women. Montgomery et al. (2012) support this finding and argue that HIV prevention, in this case through the use of female condoms, is nested within a household which is male dominated. Because female condoms are woman-controlled protective measures, their acceptability level becomes relevant in heterosexual relations. Ahmed et al. (2012) indicate that the female condom must be acceptable to both men and women in order to prevent STIs and unwanted pregnancies.

There were no statistically significant associations between contraception method and the general views about female condom. The two variables were chosen because the researchers sought to examine whether attitude as measured by the specific items might be associated with the extent of acceptability of the female condom.

Several barriers were cited such as the material of the condom, size and length, availability and insufficient marketing. A few, 4.21%, indicated that the condom can reduce sexual enjoyment. The main concern was timing of insertion. Nkobodo (2014) posit that the fact that female condom has to be inserted well in advance before sexual activity creates a hindrance to the use. The view by the majority 56.84% that the female condom is not promoted enough could reflect some fundamental challenges at the healthcare facilities and this could be a contributory factor to low usage and thus acceptability.

## Conclusion and recommendations

This study has confirmed low acceptability as manifested by low usage of female condoms in Jwaneng, Botswana. Acceptability of the female condom involves complex factors. Attitude, knowledge and power relations play a role in the extent to which women would want to use it. Level of education and marital status did not have any significant impact on the choice of contraception. Male condoms seemed to be the preferred choice. However, it is not known who initiated its use. The study concludes that women in Jwaneng had access to information or knowledge about the female condom and they were aware of the preventive benefits. However, this did not translate to increased utilisation. The study highlights the significant challenges related to of shape, material, size and low marketing of female condoms in Jwaneng. It is recommended that the Sexual-Reproductive Health unit of the Ministry of Health develop new strategies to promote the female condom, with the intent to increase utilisation in order to reduce the transmission of HIV and/or AIDS and other STIs. Involvement of men in all female condom health promotion campaigns should be ensured. Further research is needed into methods to improve the material, shape, size and insertion time.
